# Application of Various NDT Methods for the Evaluation of Building Steel Structures for Reuse

**DOI:** 10.3390/ma7107130

**Published:** 2014-10-22

**Authors:** Masanori Fujita, Tomoya Masuda

**Affiliations:** Graduate School of Science and Engineering, Yamaguchi University, 2-16-1 Tokiwadai, Ube 755-8611, Japan; E-Mail: t031vm@yamaguchi-u.ac.jp

**Keywords:** steel structural member, reuse, mechanical properties, rolled section steel, non-destructive test, hardness, chemical composition

## Abstract

The reuse system proposed by the authors is an overall business system for realizing a cyclic reuse flow through the processes of design, fabrication, construction, maintenance, demolition and storage. The reuse system is one of the methods to reduce the environmental burden in the field of building steel structures. These buildings are assumed to be demolished within approximately 30 years or more for physical, architectural, economic and social reasons in Japan. In this paper, focusing on building steel structures used for plants, warehouses and offices without fire protection, the performance of steel structural members for reuse is evaluated by a non-destructive test. First, performance evaluation procedures for a non-destructive test, such as mechanical properties, chemical compositions, dimension and degradation, are shown. Tensile strengths are estimated using Vickers hardness measured by a portable ultrasonic hardness tester, and chemical compositions are measured by a portable optical emission spectrometer. The weldability of steel structural members is estimated by carbon equivalent and weld crack sensitivity composition using chemical compositions. Finally, the material grade of structural members of the building steel structure for reuse is estimated based on the proposed procedures.

## 1. Introduction

Based on the Fourth Assessment Report of IPCC, in order to reduce the amount of anthropogenic emissions to the same level as natural absorption, it is now widely accepted to set the goal of reducing global greenhouse gas emissions by 50% by the year 2050 [1,2]. Individual countries are making a full-fledged effort by putting forward mid- and long-term scenarios and visions to address global warming.

Japan has set a long-term goal of reducing its CO_2_ emissions by 60%–80% from current levels by 2050. In order to achieve this goal, the Action Plan for Achieving a Low-carbon Society was approved in a Cabinet meeting. In 2009, based on the basic principles laid out in the charter of longer service life, natural symbiosis, energy conservation, resource conservation and cyclicity and succession, seventeen architecture-related organizations formulated “2050 Vision: Building-related Measures to Counteract Global Warming” to achieve carbon neutrality in this sector and to ultimately realize a low-carbon society [3,4]. 

**Figure 1:**
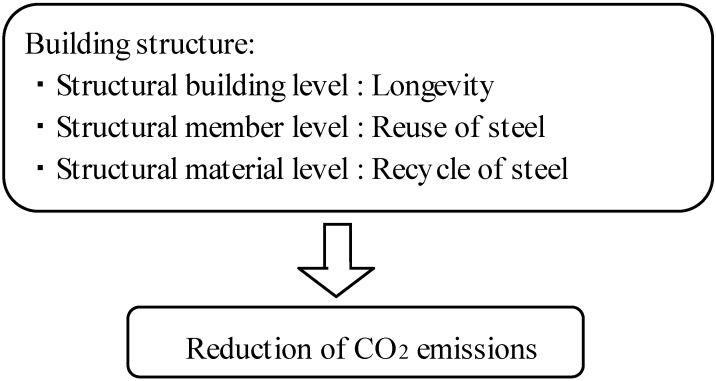
Environmental performance and building structures.

As an architectural effort to reduce the global environmental burden, the authors have been pursuing studies on evaluating the environmental burden of building steel structures, focusing on the amount of waste and life cycle CO_2_ emissions and on a reuse system for steel products. Implementing any of the following measures can be effective at reducing the environmental burden in the life cycle of building steel structures: extending the service life of buildings themselves; reuse, which is extending service life at the structural member level; and recycling, which is extending service life at the material level; shown in Figure 1 [5,6].

**Figure 2:**
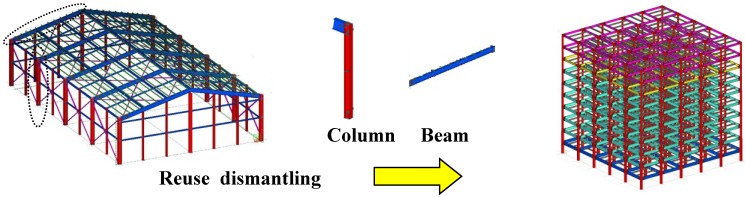
Steel structural members for reuse.

The above scenarios illustrate that extending the service life of buildings is the most crucial element in reducing the environmental burden of building steel structures. Nevertheless, there are always a number of buildings that need to be demolished for physical, architectural, economic and social reasons. When such building steel structures have been demolished in the past, their structural members have been scrapped for recycling. Scrapping steel structural members for recycling requires energy for melting, and this melting process causes substantial CO_2_ emissions. However reusing structural members requires only ancillary energy for demolition, transportation and adjustments, causing less environmental burden, shown in Figure 2 [7,8,9,10,11].

Steel, by nature, is the only type of structural member that can be refabricated. Even without special joints that facilitate demolition work, steel members can be reused after minor fabrication procedures, such as cutting, drilling and welding. Steel undergoes no major changes due to aging, except for rust and plasticization caused by large-scale earthquakes. Such excellent mechanical properties render steel suitable for reuse. Rust problems can be resolved by painting; plasticization caused by earthquakes can be handled by adopting damage-controlled design using a seismic design approach in which structural members are maintained within an elastic region by specifying seismic energy-absorbing members [12].

With respect to the performance evaluation of steel structural members for reuse, it is assumed that mechanical properties, such as tensile force, yield strength and elongation are generally evaluated by a destructive test. If test specimens can be obtained by building steel structures, it is possible to evaluate the performance by a destructive test. However, it is a difficulty to obtain a test specimen using building steel structures. Assuming building steel structures to be demolished approximately within 30 years or more, design specification and inspection certificates of building steel structures to be reused do not exist.

This paper is aiming to evaluate the performance of steel members for reuse by a non-destructive test. First, the performance evaluation procedure of steel structural members for reuse is proposed. Furthermore, based on it, steel structural performances are evaluated if they meet the Japanese Industrial Standard.

## 2. Performance of Steel Structural Members by a Non-Destructive Test

### 2.1. Steel Structural Members for Reuse

**Figure 3:**
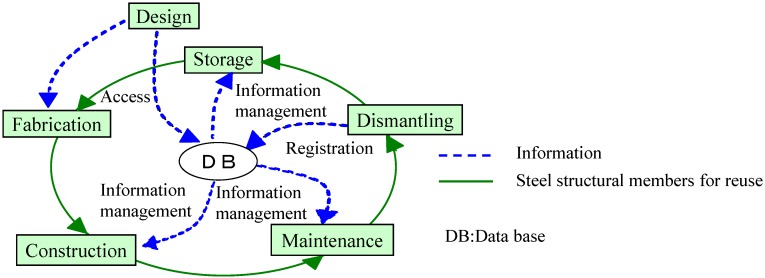
Reuse flow.

**Figure 4:**
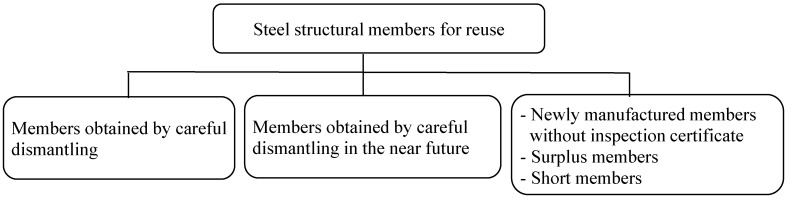
Steel structural members for reuse.

A reuse flow diagram of the building steel structure is presented in Figure 3. The dotted line shows the information flow, and the continuous line shows the flow of steel structural members for reuse. Steel structural members circulate via the database (DB) through a cyclic process: design, fabrication, construction, maintenance, dismantling and storage. The kinds of steel structural members for reuse are shown in Figure 4. The steel structural members for reuse in this study are the Japanese Industrial Standard (hereafter referred to as the JIS), such as rolled steels for general structures, rolled steel sections for welded structures and rolled steel for building structure. Bolts and other connecting materials, such as welding materials, are not included. Steel structural members for reuse are collected from non-fireproof factories, workplaces, warehouses and similar buildings pre-engineered mainly by steel manufacturers. Structural members to be reused are rolled section steels systematically sized and manufactured in accordance with JIS. Here, steel structural members except, for the weld, are evaluated, since the ductility of steel structural members varies depending on construction quality and connection detail.

### 2.2. Non-Destructive Test

Destructive tests of metallic materials, such as the tensile test, indentation test and Charpy pendulum impact test, are used to evaluate steel structural performances. On the other hand, dimensional inspection, indentation test, chemical composition test and degradation inspection can be used to evaluate structural performance as non-destructive tests. The detection of cracks in weldments using ultrasonic testing is generally used in building steel structures [13,14]. Otherwise, a method for structural parameter identification utilizing elemental strain measurement is used and successfully verified at the element level [15]. Particularly, an ultrasonic hardness tester is used in processed goods, metal molds and measuring the strength of steel sections, and it can easily measure the hardness index of materials, from the measurement of equivalent stiffness by an ultrasonic vibrator sensor, without measuring the plastic deformation [16]. A portable optical emission spectrometer is used to measure their chemical compositions in plant piping and scrap materials as a non-destructive test. If non-destructive tests can be conducted, it is possible to evaluate steel structural members for reuse that are not only obtained by reuse dismantling, but also under the use of building steel structures. Non-destructive tests can be also applied after reuse dismantling, because non-destructive tests are more economical than destructive tests.

## 3. Performance Evaluation Procedure

### 3.1. Overview

Structural steel products generally fall into four categories of JIS products, JIS equivalents, government qualified products and non-standardized products. Steel structural members for reuse in these categories are assessed for performance equivalent to newly manufactured members with inspection certificates presenting descriptions of their qualities. Performances of steel structural members are evaluated by mechanical properties (including tensile strengths, yield strengths, elongations and some others), chemical compositions, dimensions and degradation. In a destructive test, yield strengths, tensile strengths and elongations of steel structural members for reuse are conducted based on the tensile test. The tensile test is shown in the mechanical properties test of JIS Z 2241 [17]. This JIS specification corresponds to ISO 6892-1 [18]. Performance evaluations of steel structural members, such as dimensions, mechanical properties and chemical compositions by a non-destructive test, are the same with destructive tests and are shown in JIS G 3101 [19], JIS G 3106 [20], ISO 630 [21], JIS G 3136 [22], ISO 24314 [23] and some others. Weldability is evaluated by chemical analysis tests in accordance with JIS G 0321 [24] specified in ISO 10474 [25]. Steel structural members fabricated by jointing surplus and short members with an inspection certificate attached are treated as newly manufactured members, as they are fabricated in the conventional fabrication procedures.

### 3.2. Mechanical Properties and Chemical Compositions

#### 3.2.1. Tensile Strength and Hardness

There is assumed to be a positive relation between tensile strength and hardness. Tensile strength can be expressed by the following equation using Vickers hardness number [26,27].

*T*_s_ = 2.5 *H*_v_ + 100 (1)

Here, *H*_v_ is the Vickers hardness number and *T*_s_ is the tensile strength (N/mm^2^).

**Figure 5:**
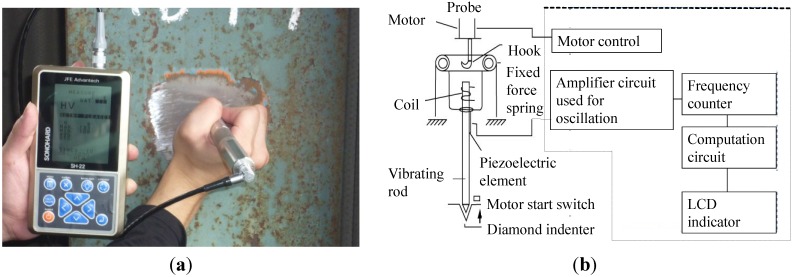
(**a**) Portable hardness tester; and (**b**) measurement principle of the portable hardness tester.

There are mainly portable ultrasonic hardness testers and rebound-type portable hardness meters in non-destructive tests. An ultrasonic hardness tester is shown in Figure 5 [28]. Instead of measuring the size of the indentation of steel using a microscope, this tester employs a diamond indenter equipped with a vibrating rod that presses on the steel surface at a fixed load and measures its hardness by applying ultrasonic vibrations and analyzing its damping effect. When the vibration rod is applied to a soft-surfaced steel with identical qualities and at a fixed force, it makes a deep indentation and gets locked into the groove. Due to this, the resonance frequency increases. Conversely, it does not get locked in when used on hard steel, and the resonance frequency drops. The steel’s hardness can be calculated using the correlation between this deviation and the tested hardness.

#### 3.2.2. Chemical Compositions

**Figure 6:**
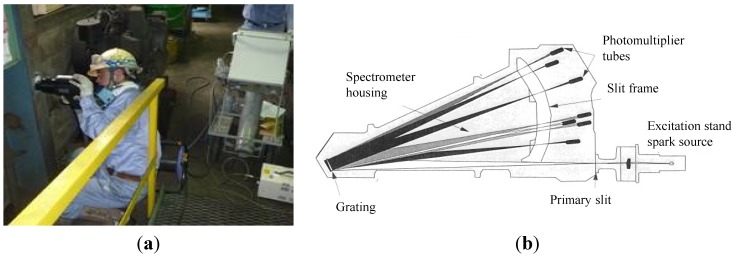
(**a**) Portable optical emission spectrometer and (**b**) the principle of portable optical emission spectrometry.

In the steel manufacturing process, an optical emission spectrometer is often used to obtain test pieces destructively, and the chemical compositions are adjusted. On the other hand, there is a portable optical emission spectrometer by a non-destructive test shown in Figure 6; materials are vaporized by a spark discharge, and the light emitted can be used for spectrometry at that time [29]. The compositions of structural members are analyzed by the wave-length, and the content of the chemical composition is analyzed by the strength on site. Carbon equivalent (C_eq_) and weld crack sensitivity composition (P_cm_) can be evaluated by chemical compositions.

On the basis of general rules for the inspection of steel (JIS G 0404) [30], the performance of steel structural members is evaluated according to product analysis and its tolerance for wrought steel (JIS G 0321 [24]). C_eq_ and P_cm_ are calculated by Equations (2) and (3), respectively [31].

*Here,*

C_eq_ = C + Mn/6 + Si/24 + Ni/40 + Cr/5 + Mo/4 + V/14 (2)

P_cm_ = C + Mn/20 + Si/30 + Cu/20 + Ni/60 + Mo/15 + V/10 + 5B (3)

### 3.3. Dimensional Evaluation

In the dimensional evaluation, the dimensions of steel structural members are measured with a slide gauge, ultrasonic device, micrometer, straight measure, angled measure and steel tape to confirm that they meet the dimensional tolerance requirements specified in the existing JIS G 3192 [32]: the shape, dimensions and mass of hot rolled H-section steels and their tolerances. For example, dimensional evaluation of rolled H-section steel is evaluated according to dimensions, mass and permissible variations of hot rolled steel sections. This JIS specification corresponds to sloping flange beam sections, dimensions and sectional properties, sloping flange column sections dimensions and sectional properties (ISO 657-15 [33], ISO 657-16 [34]).

### 3.4. Degradation Evaluation

For evaluating the paint coating degradation, the grades of blistering, rusting, cracking, flaking and chalking are categorized in JIS K 5600-8 [35] as shown in Table 1. In the JIS standards, the evaluation of paint coating degradation is standardized as testing methods for paints based on the standards specified by the International Organization for Standardization (ISO standards). In these degradation evaluation, the rating degree of blistering, rusting, cracking, flaking and chalking are evaluated by the designation of intensity, quantity and size of defects. This JIS specification corresponds to paints and vanishes (Evaluation of quantity and size of defects and of the intensity of uniform changes in appearance (ISO 4628) [36]. The thickness of painting is measured by a coating thickness tester. In evaluating paint coating degradation, if the degree of rusting is above Grade 4 (Ri4 (S4)), the rust is removed by shot blasting (JIS K 5600-8-3 [35], ISO 4628-3 [36]). The coating thickness is measured with an electromagnetic thickness gauge.

**Table 1:** JIS, ASTM, ISO specification of paint coating degradation.

JIS Specification (Paint coating degradation evaluation)	ASTM Specification	ISO Specification
JIS K 5600-8-1 (General rules and grades) [35]	–	ISO 4628-1 [36]
JIS K 5600-8-2 (Grade of blistering) [35]	ASTM D 714-02 [37]	ISO 4628-2 [36]
JIS K 5600-8-3 (Grade of rusting) [35]	ASTM D 610-01 [38]	ISO 4628-3 [36]
JIS K 5600-8-4 (Grade of cracking) [35]	–	ISO 4628-4 [36]
JIS K 5600-8-5 (Grade of flaking) [35]	–	ISO 4628-5 [36]
JIS K 5600-8-6 (Grade of chalking) [35]	–	ISO 4628-6 [36]

### 3.5. Evaluation Flow

**Figure 7:**
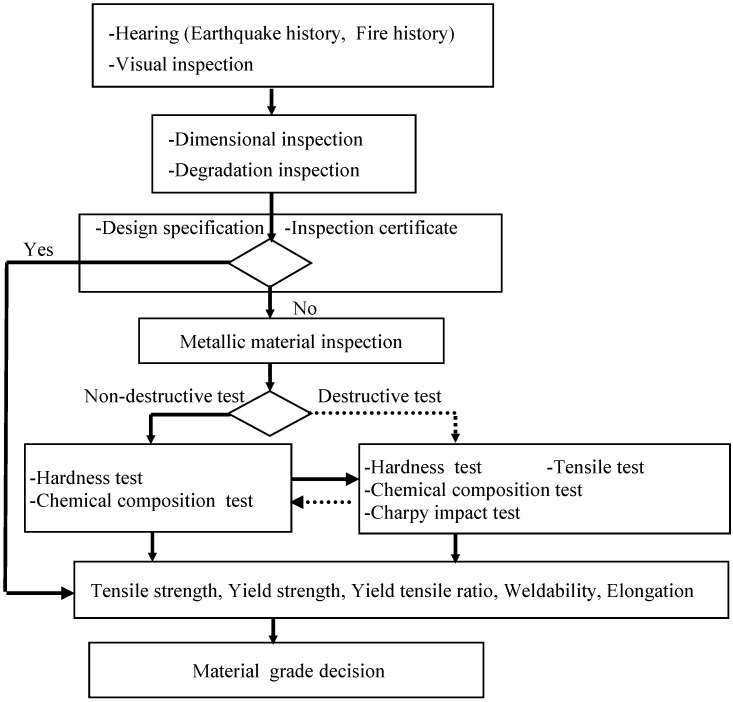
Evaluation flow of steel structural members for reuse.

The evaluation flow of steel structural members for reuse is shown in Figure 7. First, damages of structural members, such as earthquake history, fire history according to the hearing from the owner, design specification and the inspection certificate of the building structure are estimated. Secondly, visual inspection is conducted to determine whether structural members are adequate for reuse before dismantling. If structural members are approximately evaluated for reuse, dimensional inspection and degradation inspection are conducted. Thirdly, if design specification or an inspection certificate exist, structural members can be evaluated the same as newly manufactured members. If design specification or an inspection certificate does not exist, metallic material inspections are conducted by destructive or non-destructive tests. If test pieces can be obtained by building steel structures, structural members are evaluated by destructive tests. There are primarily used destructive tests, such as the tensile test, bending test and Charpy pendulum impact test. However, if test pieces cannot be obtained because building structures are in use, structural members should be evaluated by non-destructive tests, such as the ultrasonic hardness test and chemical composition test. Generally, non-destructive tests are more economical than destructive tests. If performance evaluation methods of steel structural members by non-destructive tests are established, it will help steel structural members be reused. Particularly, elongation evaluation by a non-destructive test is a challenge for the future. If elongations of steel structural members are evaluated, these members will be used for main building steel structures to enable plastic deformations for reuse. Finally, based on tensile strength, yield strength, member properties and weldability are evaluated. A combination of destructive tests and non-destructive tests also can be used.

## 4. Performance Evaluation of Building Steel Structures

Based on the procedures mentioned in Section 3, the performances of structural member for reuse are evaluated using a practical building steel structure.

### 4.1. Overview

**Figure 8:**
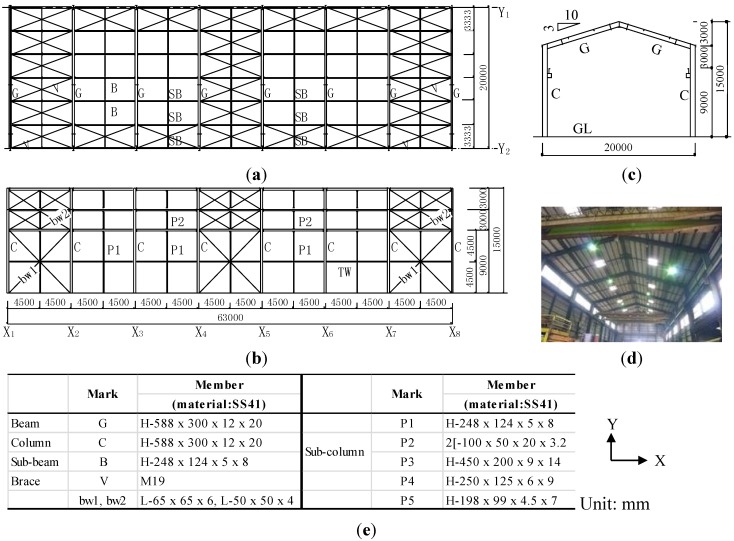
(**a**) Plan; (**b**) elevation (y-axis); (**c**) elevation (x-axis); (**d**) interior view; and (**e**) the members’ list of the building steel structure.

This building steel structure is a factory using rolled H-section steel, and these members have non-proofing protection. This building structure is located less than 2 km from the coast. It was completed in 1974, and the owner had changed. Design specification exists, but an inspection certificate does not exist. According to the owners, there are no damages, such as earthquakes, typhoons or fires, in the past. The roof plan, the elevation x- and y-axes and interior view are shown in Figure 8a–d. The members’ list is also shown in Figure 8e. This building steel structure uses a one-way brace structure and a one-way rigid frame structure (using two three-ton cranes) with a span length of 20 m × 63 m and an eave height of 12 m. The sizes of the beams and columns are H-588 × 300 × 12 × 20. The beam to column connection of the building steel structure is a bracket type with welding. The columns, beams, sub-beams and sub-columns are connected with high strength bolts.

### 4.2. Mechanical Properties

**Figure 9:**
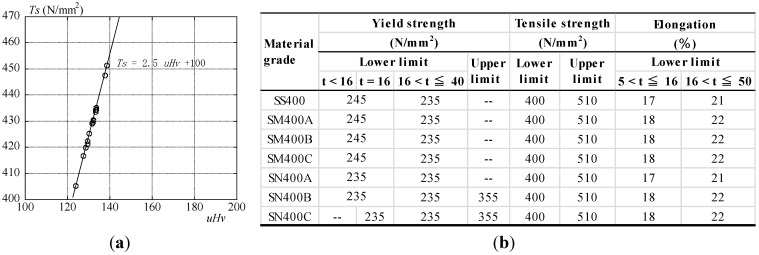
(**a**) Tensile strength and hardness and (**b**) mechanical properties of the material grade of JIS.

Tensile strength and hardness *_u_H_v_* measured by the ultrasonic hardness tester are shown in Figure 9a. Tensile strengths are calculated to Vickers hardness numbers using Equation (1). Columns of steel structural members for the measurement location are chosen as representative. The thickness of the flange of the column is 20 mm. Before Vickers hardness numbers are measured, the surfaces of steel members are ground. A three-times Vickers hardness value is measured as the average per measurement point. *_u_H_v_* has a liner relation to tensile strength and is distributed over 405–451 N/mm^2^ in Figure 9a. The average of *_u_H_v_* is 132 (standard deviation = 3.5). 

On the other hand, the tensile strength of the specimen of the newly manufactured steel prepared in advance by the destructive tensile test is 439 N/mm^2^ (average of three specimens). The ratio tensile strength by the destructive tensile test to the corresponding value by *_u_H_v_* is approximately 95%. This value is close to the corresponding value measured by the ultrasonic hardness tester. The mechanical properties of steel members correspond to SS400 (400 N/mm^2^ grade steel) for JIS and are shown in Figure 9b. The lower limits of yield strength, tensile strength and elongation are classified by the thickness (t) of steel structural members. The lower limit and upper limit of tensile strength, the lower limit of the yield strength and elongation for 400 N/mm^2^ grade steel are defined. As the tensile strength by the corresponding value measured by the ultrasonic tester is 400–510 N/mm^2^, all of the material grades correspond to the value of JIS. Since this building was completed in 1974, SN400 does not correspond to this one, so as to be standardized in 1994. Though elongation evaluation is necessary for further study, SS400 is the lowest one in the lower limit of elongation in SS400, SM400A, SM400B and SM400C. For this reason, as this material grade is assumed to be SS400, considered safe, it is consistent with the design specification. From the above results, the tensile strength of the steel structural members is approximately assumed by hardness using an ultrasonic hardness tester.

### 4.3. Chemical Compositions

**Figure 10:**
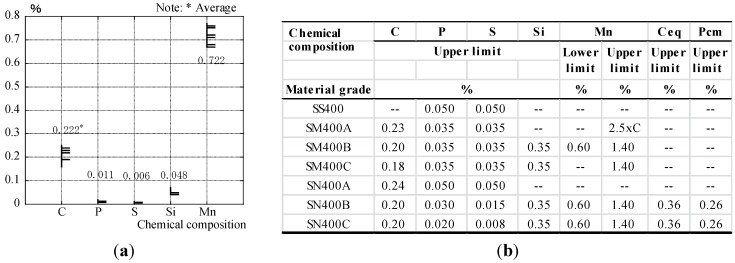
(**a**) Chemical composition and (**b**) chemical compositions of the material grade of JIS.

**Figure 11:**
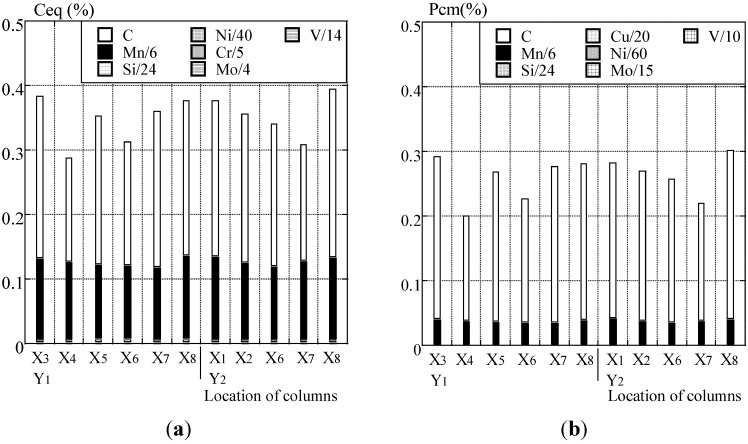
(**a**) Carbon equivalent (C_eq_) and (**b**) weld crack sensitivity composition (P_cm_).

The distribution of chemical compositions of C, P, S, Si and Mn measured by the non-destructive test is shown in Figure 10a. Before the chemical compositions of steel structural members are measured, the surfaces of the flange are ground the same as for the ultrasonic hardness tester. The chemical compositions of steel members corresponding to SS400 (400 N/mm^2^ grade steel) for JIS are shown in Figure 10b. Carbon (C), phosphorus (P) and sulfur (S) of each column are distributed as 0.16%–0.26%, 0.005%–0.017% and 0.001%–0.015% respectively. C_eq_ and P_cm_ are shown by chemical composition in Figure 11a,b, respectively. Copper (Cu: 0.01%), molybdenum (Mo: 0.01%) and vanadium (V: 0.002%) are minimum values, which can be measured by a non-destructive test.

In calculating P_cm_, Boron (B) is assumed to be zero, because it cannot be measured. C_eq_ and P_cm_ are dominated by carbon (C) and manganese (Mn) according to chemical compositions. C_eq_ and P_cm_ are distributed as 0.28%–0.39% and 0.19%–0.30% respectively. As the chemical compositions of P and S measured by the non-destructive test meet the upper limit, all of the material grades correspond to the value of JIS. The maximum composition of C is 0.26%, though it has variation in the measured point. It only corresponds to SS400 without the upper limit value. Almost all of the measurements in C_eq_ and P_cm_ do not meet SN400B and SN400C with the upper limit. The mechanical properties of steel structural members are assumed to be SS400 grade steel by chemical compositions, and they are consistent with the design specification.

With respect to the above results, the weldability of steel structural members is assumed by carbon equivalent (C_eq_) and weld crack sensitivity composition (P_cm_) by the non-destructive test.

### 4.4. Dimension and Degradation

**Figure 12:**
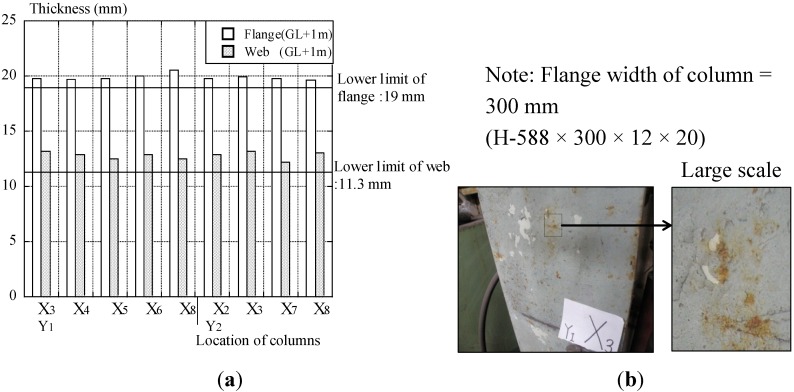
(**a**) Thickness of the flange and web on the column and (**b**) rusting of the Y_1_X_3_ column (Ri3(S5)).

The thickness of the flange and web on the column is shown in Figure 12a. In dimensional inspection, the thickness of the flange and web (height of measurement: ground level + 1 m) is measured by an ultrasonic device or vernier. Three times the measured values are averaged per point. The thickness of the web mainly is measured by an ultrasonic thickness device, because it cannot be measured by the vernier. This thickness of columns meets the 1-mm allowance in the flange and 0.7 mm in the web (JIS G 3192) [32].

Maintenance of the paint coating of structural members was conducted a few times in the past, and the measured paint coating thickness of the columns was distributed over 26~250 μm. The rust of structural members is often found near the entrance, but the thickness of these members is not decreased. The reasons are because steel structural members are used for indoors, and the requirement of chemical resistance performance is not necessary. Almost all of the degradation of steel structural members for rusting corresponds to Ri3(S4) or Ri3(S5), shown in Figure 12b.

According to visual inspection, large damage of structural members was not found, and it is assumed that there has not been any history of damage, such as earthquakes and fires, as the result of the hearing from the owner.

## 5. Conclusions

This paper examined the performance evaluation of steel structural members for reuse using a non-destructive test. The following results are obtained:

(1) A performance evaluation procedure of steel structural members for reuse is proposed using a non-destructive test, and the evaluation flow of steel structural members is shown.
(2) The mechanical properties of steel structural members are assumed to be SS400 grade steel by hardness or chemical compositions in the non-destructive test and meet the design specification.
(3) The tensile strengths of steel structural members are assumed by hardness using an ultrasonic hardness tester.
(4) Based on the performance evaluation procedure of steel structural members, the performances of structural members of the building steel structure for reuse are verified.
